# Supplementary material for: Extracellular PKM2 Preserves Cardiomyocytes and Reduces Cardiac Fibrosis During Myocardial Infarction
Source: Int J Mol Sci. 2024 Dec 10;25(24):13246. doi: 10.3390/ijms252413246 (PMC11675365; doi:10.3390/ijms252413246)
Supplement: Supplementary file 1 [file ijms-25-13246-s001.zip › ijms-3325326-supplementary.pdf]

Extracellular PKM2 Preserves Cardiomyocytes and Reduces Cardiac Fibrosis during Myocardial Infarction

Yang Huang[1], Bin Li[1], Zongxiang Gui[2], Erhe Gao[3], Yi Yuan[1], Jenny Yang[2], Nedumangalam Khan Hekmatyar[2], Falguni Mishra[1], Payton Chan[1], and Zhi-Ren Liu[1*]

[1]Department of Biology, [2]Department of Chemistry
Georgia State University, Atlanta, GA 30303, USA
[3]Center for translational medicine, Temple University
Philadelphia, PA, 19140 USA.

* Corresponding author

Zhi-Ren Liu, Ph.D.
Department of Biology
Georgia State University
University Plaza
Atlanta, GA 30303 USA
(zliu8@gsu.edu)

Materials

Table S1. Chemicals, Reagents and Solutions.

Reagent or Resource	Source	Identifier
ImmEdge®Hydrophobic Barrier PAP Pen	Vector Laboratories	H-4000
VECTABOND® Reagent	Vector Laboratories	SP-1800-7
Antigen Unmasking Solution, Citrate-Based	Vector Laboratories	H-3300-250
Vector® TrueVIEW® Autofluorescence Quenching Kit	Vector Laboratories	SP-8500-15
Animal-Free Blocker®, 5X Concentrate	Vector Laboratories	SP-5030-250
BLOXALL® Endogenous Blocking Solution	Vector Laboratories	SP-6000-100
Animal-Free Blocker® and Diluent, RTU	Vector Laboratories	SP-5035-100
MACH 2 Rabbit HRP-Polymer	Biocare Medical	RHRP520L
MACH 2 Mouse HRP-Polymer	Biocare Medical	MHRP520L
ImmPRESS®-AP Horse Anti-Rabbit IgG Polymer Detection Kit	Vector Laboratories	MP-5401-15
ImmPRESS®-AP Horse Anti-Mouse IgG Polymer Detection Kit	Vector Laboratories	MP-5402-15
ReadyProbes™ Mouse-on-Mouse IgG Blocking Solution (30X)	Thermo Fisher Scientific	R37621
Hematoxylin Counterstain	Vector Laboratories	H-3401-500
ImmPACT® Vector® Red Substrate Kit, Alkaline Phosphatase (AP)	Vector Laboratories	SK-5105
ImmPACT® DAB Substrate Kit, Peroxidase (HRP)	Vector Laboratories	SK-4105
Protein inhibitor cocktail (100X)	Thermo Fisher	87786
Phosphatase inhibitor cocktail I (100X)	Thermo Fisher	78420
RIPA lysis buffer	Thermo Fisher	89900
Protein Ladder	Thermo Fisher	26634
ECL Western Blotting substrate	Thermo Fisher	32106
Western Blot Stripping Buffer	Thermo Fisher	46430
Flow cytometry Staining Buffer	Thermo Fisher	00-4222-26
ProLong™ Diamond Antifade Mountant with DAPI	Thermo Fisher	P36962
Tris Base	Fisher Scientific	BP154-1
Tween-20	Sigma-Aldrich	P5927
Xylenes	Fisher Scientific	X5-4
Ethanol	Decon Lab	22032601
Molecular Biology Agarose	Bio-Rad	1613101
FAK Inhibitor 14	Sigma Aldrich	SML0837-10MG
LY-294,002	Sigma Aldrich	L9908
Triton X-100	Sigma Aldrich	X100-500ML
Wheat Germ Agglutinin, Alexa Fluor™ 488 Conjugate	Thermo Fisher	W11261
IPTG, dioxane-free	Thermo Fisher	R0392
DMEM, high glucose, no glutamine, no phenol red	Thermo Fisher	31053028
Sodium Pyruvate (100 mM)	Thermo Fisher	11360070
L-Glutamine (200 mM)	Thermo Fisher	25030081
HyClone Characterized Fetal Bovine Serum (FBS), US Origin	Cytiva	SH30071.03
Cardiac Myocyte Medium Phenol Red Free (includes supplements)	SCIENCELL	6201-prf
Poly-L-Lysine, 1 mg/ml	SCIENCELL	0403
Recombinant Human PKM2 His Protein	Novus Biologicals	NBP1-30292

Table S2. Antibodies.

Antibodies	Source	Identifier
Goat anti-Mouse IgG (H+L) Cross-Adsorbed ReadyProbes™ Secondary Antibody, Alexa Fluor™ 594	Thermo Fisher	R37121
Goat anti-Mouse IgG (H+L) Cross-Adsorbed ReadyProbes™ Secondary Antibody, Alexa Fluor™ 488	Thermo Fisher	R37120
Goat anti-Rabbit IgG (H+L) Cross-Adsorbed ReadyProbes™ Secondary Antibody, Alexa Fluor™ 594	Thermo Fisher	R37117
Goat anti-Rabbit IgG (H+L) Cross-Adsorbed ReadyProbes™ Secondary Antibody, Alexa Fluor™ 488	Thermo Fisher	R37116
Goat anti-Rabbit IgG (H+L) Cross-Adsorbed Secondary Antibody, HRP	Thermo Fisher	G-21234
Goat anti-Mouse IgG (H+L) Cross-Adsorbed Secondary Antibody, HRP	Thermo Fisher	G-21040
PKM2 (D78A4) XP® Rabbit mAb	Cell Signaling	4053
PKM1 (D30G6) XP®Rabbit mAb	Cell Signaling	7067
Beta-Actin Monoclonal Antibody	Yurogen	R15006MC4
CD61 (Integrin beta 3) Recombinant Rabbit Monoclonal Antibody (SJ19-09)	Thermo Fisher	MA532077
Anti-Integrin αVβ3 Antibody, clone LM609	Sigma Aldrich	MAB1976
Integrin alpha V beta 3 Antibody (SC56-07)	Novus Biologicals	NBP2-67557
CD51/CD61 Mouse anti-Human, Unlabeled, Clone: 23C6, BD	BD Biosciences	555504
Alexa Fluor™ 594 Phalloidin	Thermo Fisher	A12381
Alpha-Smooth Muscle Actin Monoclonal Antibody (1A4),	eBioscience	14-9760-82
Ki-67 Recombinant Rabbit Monoclonal Antibody (SP6)	Thermo Fisher	MA514520
Cardiac Troponin T Monoclonal Antibody (13-11)	Thermo Fisher	MA512960
Troponin I Type 3 (cardiac) Antibody (4C2cc)	Novus Biologicals	NB110-8453
Phosphor PI3K	Thermo Fisher	PA5-17387
Phosphor FAK	Cell signaling	3283
α-SMA (Mouse)	Sigma-Aldrich	A5228
α-SMA (Rabbit)	Abcam	AB5694
Anti-PTEN Antibody, clone 6H2.1	Sigma Aldrich	04-035
Cell Signaling Technology PI3 Kinase p85	Cell Signaling	4292S
FAK Antibody	Cell Signaling	3285S
Cleaved PARP (Asp214) (D64E10) XP® Rabbit mAb	Cell Signaling	5625S
Cleaved Caspase-3 (Asp175) (5A1E) Rabbit mAb	Cell Signaling	9664S
Caspase-3 Antibody #9662	Cell Signaling	9662S
PARP (46D11) Rabbit mAb #9532	Cell Signaling	9532S

Table S3. Commercial Assay kits.

Kit	Source	Identifier
PI3 Kinase Activity/Inhibitor ELISA	Sigma Aldrich	17-493
Masson's trichrome kit	IHC world	IW-3006
Click-iT™ EdU Cell Proliferation Kit for Imaging	Thermo Fisher	C10337
LIVE/DEAD™ Viability/Cytotoxicity kit	Thermo Fisher	L3224
LIVE/DEAD™ Cell Imaging Kit	Thermo Fisher	R37601
Mouse Pyruvate Kinase, Muscle (PKM2) ELISA Kit	Biomatik Corporation	EKU06995
Human PKM2 ELISA Kit	Mybiosource	MBS2505089

Mouse Troponin I Type 3 (cardiac) ELISA Kit (Colorimetric)	Novus Biologicals	NBP3-00456
Lipofectamine 3000 Transfection Reagent	Thermo Fisher	L3000001
ImmPRESS® Duet Double Staining Polymer Kit	Vector Laboratories	MP-7714-15
Click-iT™ Plus TUNEL Assay Kits for In Situ Apoptosis Detection	Thermo Fisher	C10617

Table S4. Cell Lines.

Cell lines	Source	Identifier
H9C2(2-1)	ATCC	CRL-1446™
Human Cardiac Myocytes	SCIENCELL	6200

Supplementary Figures and Legends

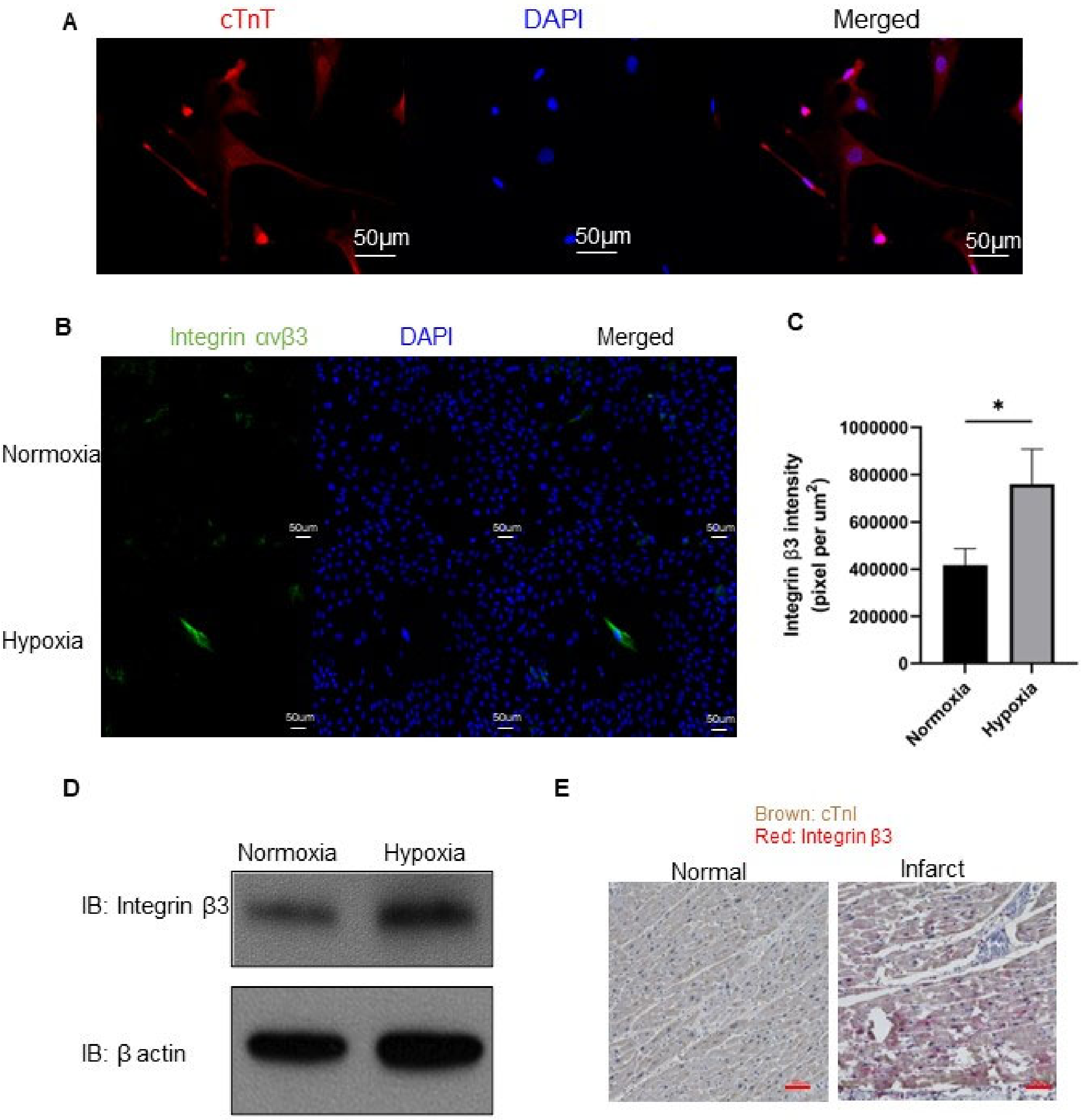

Figure. S1. Expression of integrin $\alpha_v\beta_3$ in cardiomyocyte.

(A) Representative images of IF staining of cardiac troponin T (cTnT) in commercial cardiomyocytes. **(B) and (C)** Representative images (B) and quantification (C) of IF staining of integrin $\alpha_v\beta_3$ in H9C2 cells cultured under normoxia or hypoxia conditions. The quantity in (C) is presented as integrin $\alpha_v\beta_3$ staining intensity per view field**. (D)** Immunoblot of integrin β3 in extracts prepared from H9C2 cells cultured under normoxia and hypoxia conditions. Immunoblot of β-actin is a loading control. **(E)** Representative image of IHC staining of integrin β3 in myocardium tissue of infarction patients ($n = 4$). Normal means the tissue samples from non-infarction region, while infarction indicates the tissue samples from infarction region. Error bars in (B) represent mean ± S.E.M.. Scale bars in (A) and (B) 50 μm and in (E) are 100 μm. ns statistical no-significance, * $P > 0.05$, ** $P > 0.01$, *** $P > 0.005$, **** $P > 0.001$.

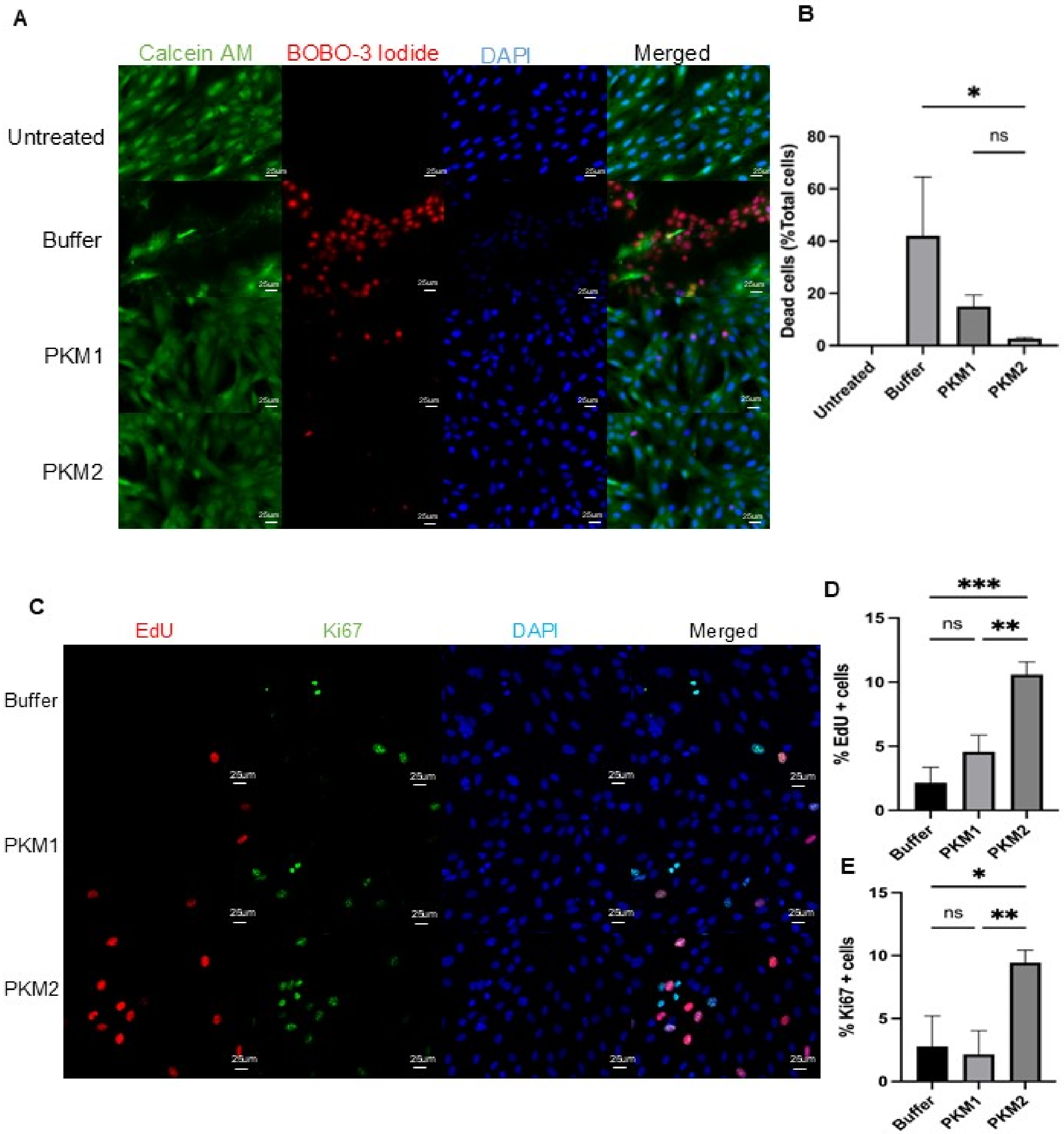

A
Calcein AM
BOBO-3 Iodide
DAPI
Merged
Untreated
Buffer
PKM1
PKM2
B
Dead cells (%Total cells)
80
60
40
20
0
*
ns
Untreated
Buffer
PKM1
PKM2
C
EdU
Ki67
DAPI
Merged
Buffer
PKM1
PKM2
D
% EdU + cells
15
10
5
0
***
ns
**
Buffer
PKM1
PKM2
E
% Ki67 + cells
15
10
5
0
*
ns
**
Buffer
PKM1
PKM2

Figure. S2 EcPKM2 protects cardiomyocytes and promotes cardiomyocyte proliferation *in vitro* under hypoxia. **(A) and (B)** Representative images (A) and quantification (B) of Calcein AM and iodide staining of *H9C2 cells* cultured under normoxia (untreated) and hypoxia conditions. The cultured cells were treated by the indicated agents. The quantity (B) is presented as % of dead cells in the total cell population. **(C) – (E)** Representative images (C) and quantification (D) & (E) of EdU and Ki67 staining of *H9C2 cells* cultured under hypoxia conditions. The cultured cells were treated by the indicated agents. The quantities in (D) and (E) are presented as % of EdU (D) or Ki67 (E) positive cells in total cell population. Error bars in (B), (D), and (E) represent mean ± S.E.M.. Scale bars in (A) and (C), 50 μm and (F) 100 μm. ns statistical no-significance, * P > 0.05, ** P > 0.01, *** P > 0.005, **** P > 0.001.

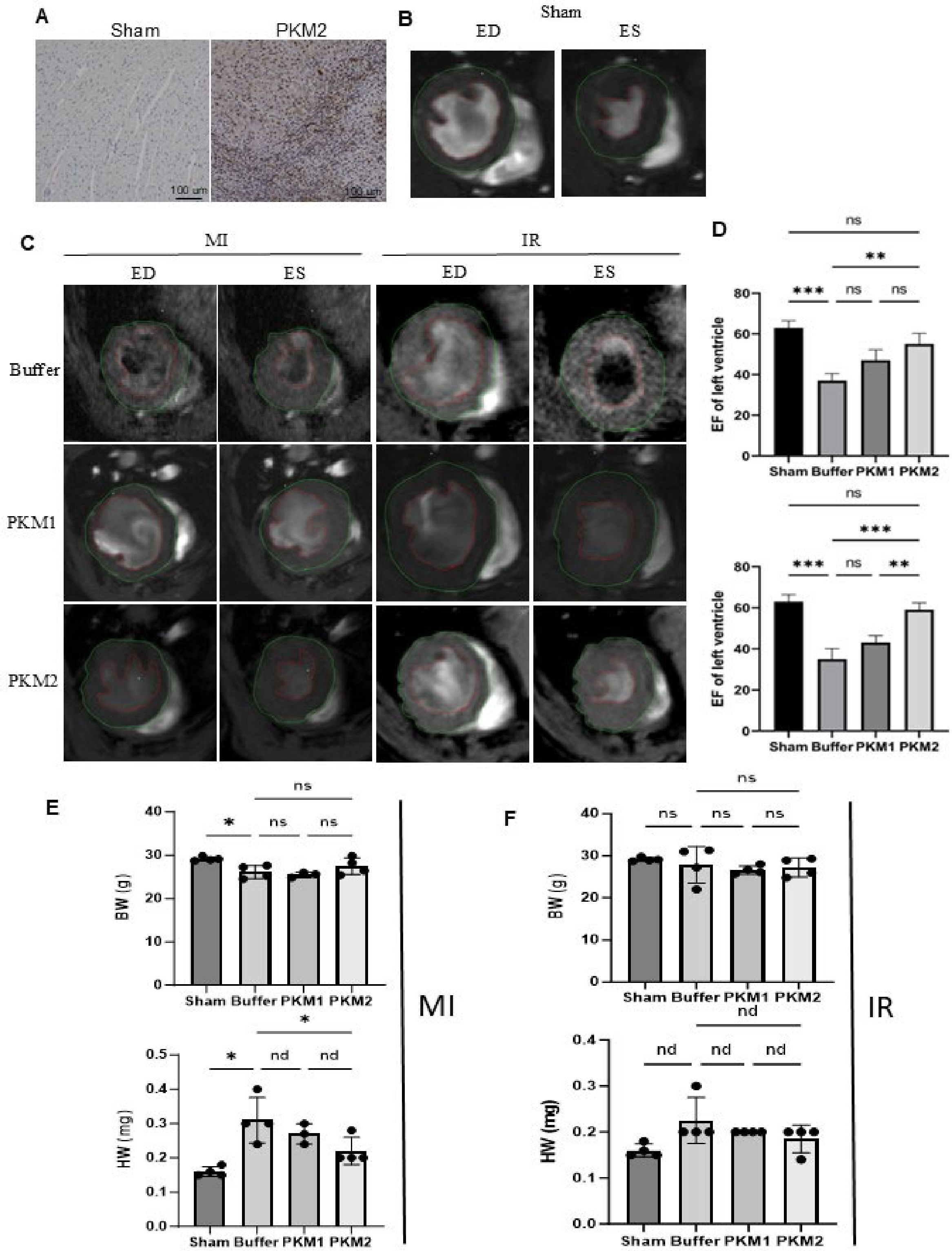

A
Sham
PKM2
100 um
100 um
B
Sham
ED
ES
C
MI
IR
ED
ES
ED
ES
Buffer
PKM1
PKM2
D
ns
**
***
ns
ns
EF of left ventricle
Sham Buffer PKM1 PKM2
ns
***
***
ns
**
EF of left ventricle
Sham Buffer PKM1 PKM2
E
ns
*
ns
ns
BW (g)
Sham Buffer PKM1 PKM2
*
*
nd
nd
HW (mg)
Sham Buffer PKM1 PKM2
MI
F
ns
ns
ns
ns
BW (g)
Sham Buffer PKM1 PKM2
nd
nd
nd
nd
HW (mg)
Sham Buffer PKM1 PKM2
IR

Figure. S3 MRI monitor effects of G415R on infarction heart. **(A)** Representative images of His-Tag IHC staining of myocardium from myocardial infarction mice 4 days after infarction induction and G415R treatment. **(B) & (C)** Representative MR images of cardiac ES and ED from sham mice (B) and mice that induced MI/IR infarction and under indicated treatments (C). **(D)** Cardiac ejection fraction of infarction MI (Upper panel) and IR (Lower panel) mice as compared to sham mice calculated from the MR imaging (n = 4). **(E) & (F)** Body weight (Upper) or heart weight (Bottom) of infarction MI (E) and IR (F) mice 30 days after infarction induction (n = 4). The mice underwent indicated treatments. Error bars in (D), (E), and (F) represent mean ± S.E.M.. Scale bars in (A) 100 μm. ns statistical no-significance, ** P > 0.01, *** P > 0.005, **** P > 0.001.

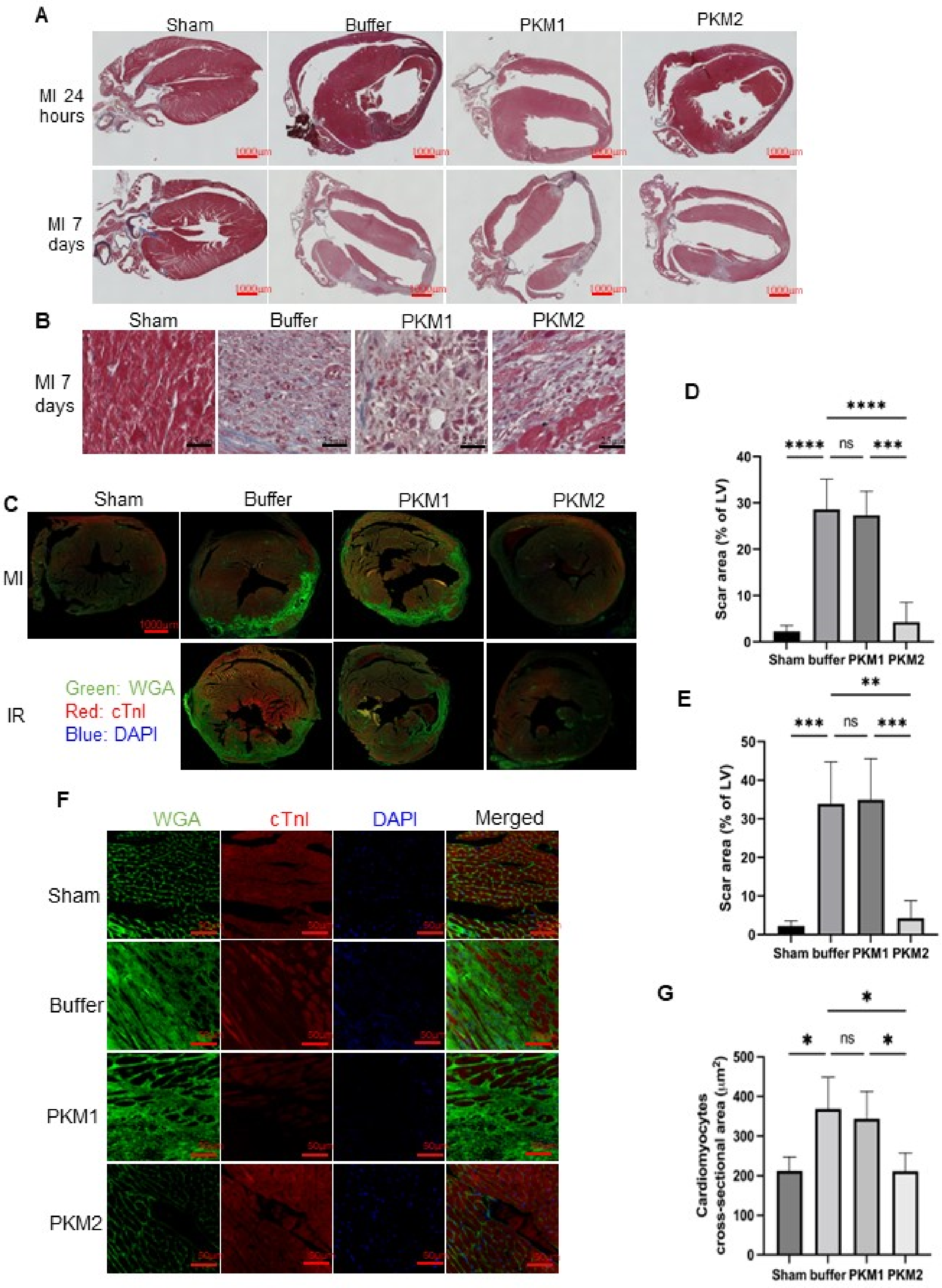
A
Sham
Buffer
PKM1
PKM2
MI 24 hours
MI 7 days
B
Sham
Buffer
PKM1
PKM2
MI 7 days
C
Sham
Buffer
PKM1
PKM2
MI
IR
Green: WGA
Red: cTnI
Blue: DAPI
D
Scar area (% of LV)
Sham buffer PKM1 PKM2
E
Scar area (% of LV)
Sham buffer PKM1 PKM2
F
WGA
cTnI
DAPI
Merged
Sham
Buffer
PKM1
PKM2
G
Cardiomyocytes cross-sectional area (μm²)
Sham buffer PKM1 PKM2

Figure. S4 EcPKM2 reduces infarction scar size and myocardium hypertrophy.

(A) & **(B)** Representative images of Masson Trichrome staining of series myocardium tissue sections (vertical section) from infarction mice 7 days after infarction induction. The images in (B) are magnified images to show details of myocardium structure. **(C) - (E)** Representative images (C) and quantification (D, MI and E, IR) of Wheat germ agglutinin (WGA) staining (green) and cTnI IF staining (red) of myocardium tissues from MI (top panel in C) and IR (bottom panel in C) infarction mice 30 days after infarction. The quantities in (D) and (E) are expressed as % of WGA positive areas in the left ventricle (Scar area % of LV). **(F)** Representative magnified images WGA (green) or cTnI IF (red) staining of myocardium tissue (horizontal section) from infarction IR mice 30 days after infarction induction. **(G)** Quantification of cardiomyocyte cross-sectional area of the infarction IR hearts of mice that treated with indicated agents and 30 days after infarction induction. Quantities are presented as cross-sectional area in μm. Error bars in (D), (E), and (G) represent mean ± S.E.M.. Scale bars in (A) and (C), 1000 μm, in (B), 25 μm, and in (F) are 50 μm. ns statistical no-significance, * $P > 0.05$, ** $P > 0.01$, *** $P > 0.005$, **** $P > 0.001$.

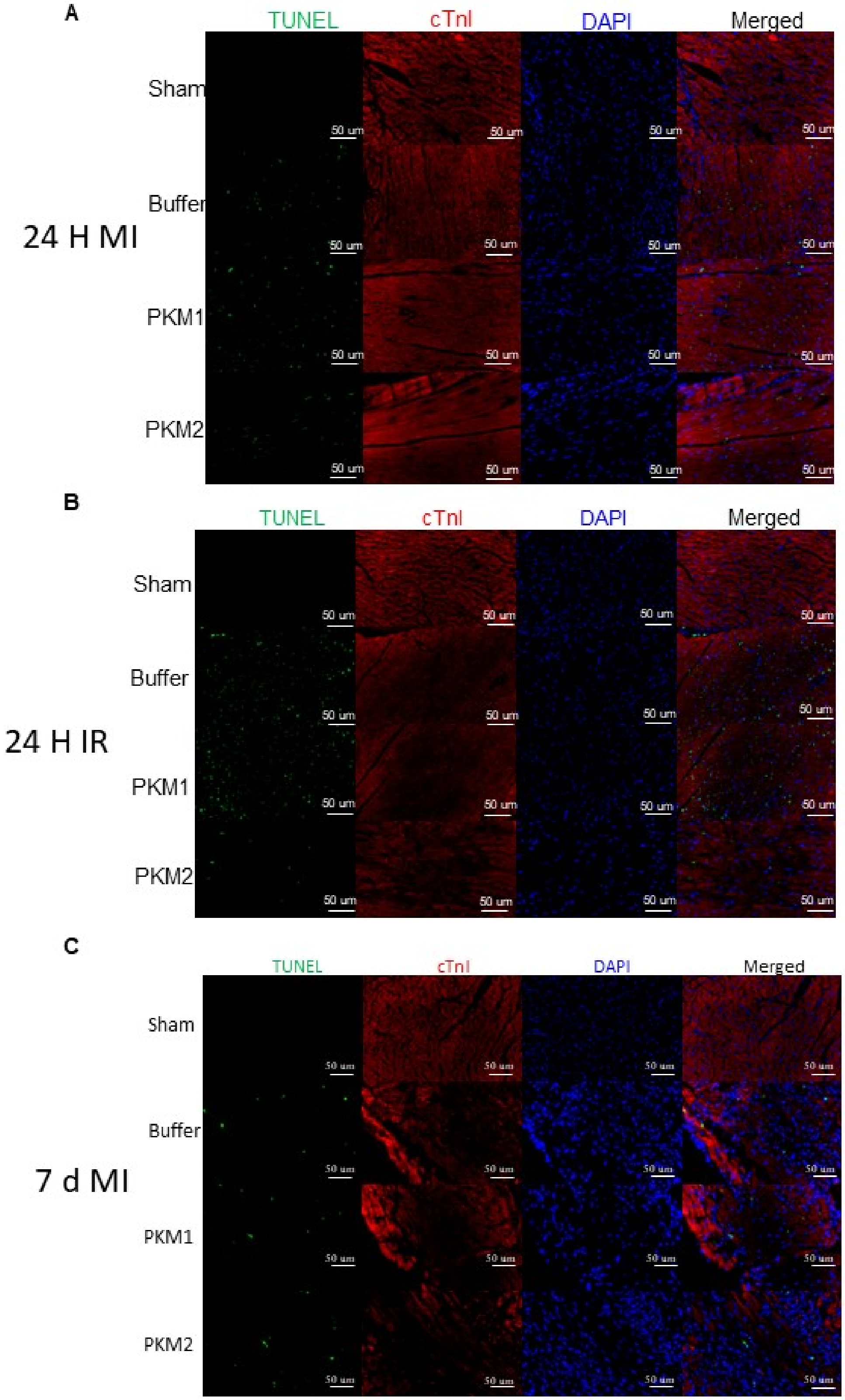
A
TUNEL
cTnI
DAPI
Merged
24 H MI
Sham
Buffer
PKM1
PKM2
50 um
B
TUNEL
cTnI
DAPI
Merged
24 H IR
Sham
Buffer
PKM1
PKM2
50 um
C
TUNEL
cTnI
DAPI
Merged
7 d MI
Sham
Buffer
PKM1
PKM2
50 um

Figure. S5 EcPKM2 protects cardiomyocytes from apoptosis and promotes proliferation in myocardium of the infarction heart.

(A), (B), & **(C)** Representative images of TUNEL (green) or cTnI IHC (red) staining of myocardium tissue from infarction mice. The animals were euthanized 24 hours (A) & (B), or 7 days (C) after infraction (MI, A and C) or 24 hours after infarction (IR, B). The animals were treated by the indicated agents. Sham mice were without ligation and without treatment. Scale bars are 50 μm.

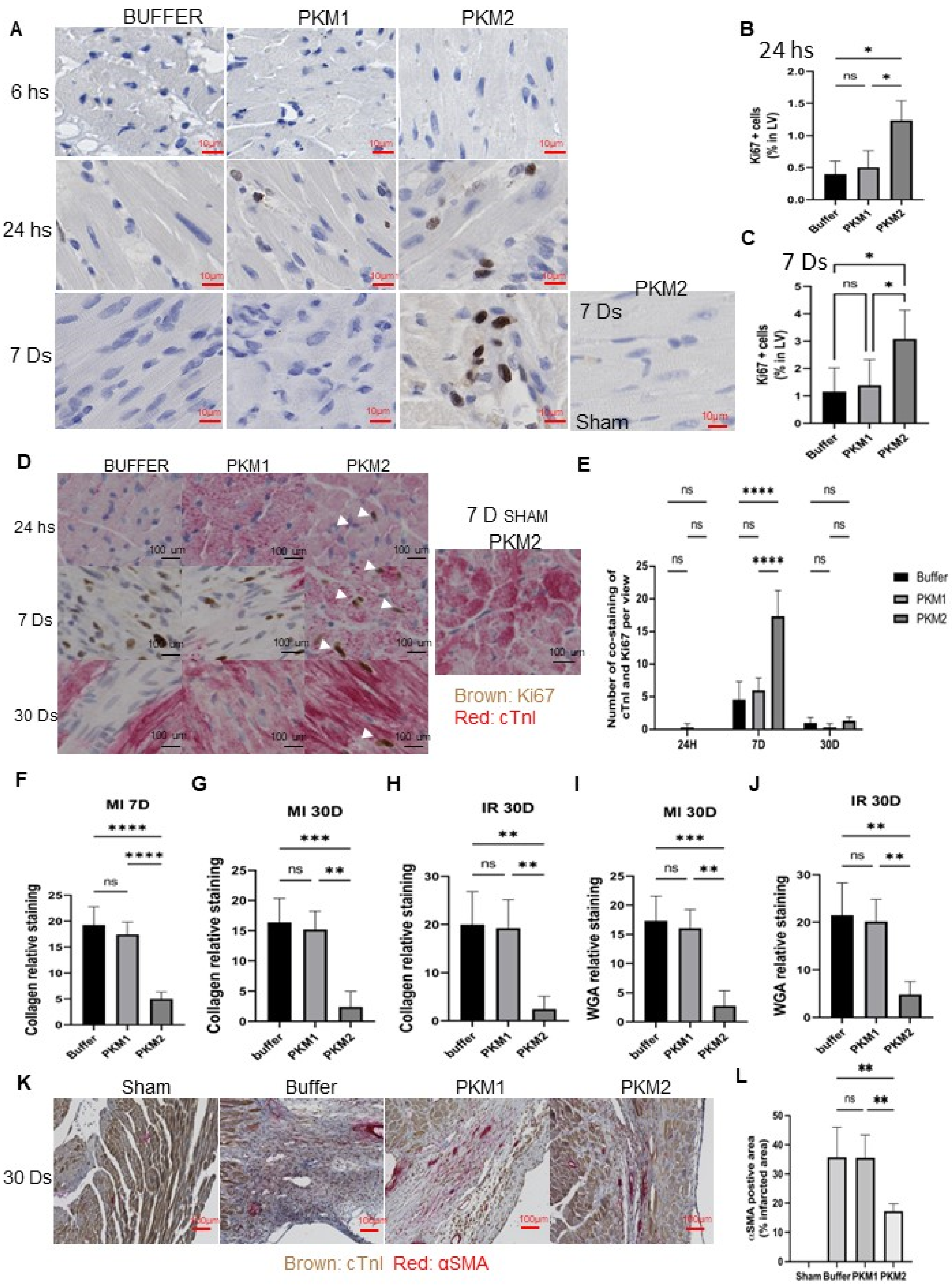
A
BUFFER
PKM1
PKM2
6 hs
24 hs
7 Ds
PKM2
7 Ds
Sham
10μm
B
24 hs
Ki67 + cells (% in LV)
Buffer
PKM1
PKM2
ns
*
C
7 Ds
Ki67 + cells (% in LV)
D
BUFFER
PKM1
PKM2
24 hs
7 Ds
30 Ds
100 um
7 D SHAM
PKM2
Brown: Ki67
Red: cTnI
E
Number of co-staining of cTnI and Ki67 per view
24H
7D
30D
Buffer
PKM1
PKM2
****
F
MI 7D
Collagen relative staining
G
MI 30D
Collagen relative staining
buffer
H
IR 30D
Collagen relative staining
I
MI 30D
WGA relative staining
J
IR 30D
WGA relative staining
K
Sham
Buffer
PKM1
PKM2
30 Ds
100μm
Brown: cTnI Red: αSMA
L
αSMA postive area (% infarcted area)
Sham
Buffer
PKM1
PKM2

Figure. S6 EcPKM2 protects cardiomyocytes from apoptosis and promotes proliferation in myocardium of the infarction heart.

(A) Representative images of Ki67 IHC staining of myocardium tissue of infarction region from the infarction mice. **(B) & (C)** Quantification of the Ki67 IHC staining. The quantity is presented as % of Ki67 positive cells in the whole left ventricle (LV). The animals were euthanized at indicated time points after infraction (B, 24 hours after MI, or C, 7 days after MI). The animals were treated by indicated agents in A - C. **(D)** & **(E)** Representative images (D) and quantification (E) of IHC Ki67 (Brown) and cTnI (Red) co-staining of myocardium tissue from infarction mice that were treated with indicated agents. The tissue samples were collected at the indicated time points of post-infarction. The quantity in (E) is presented as number of Ki67 and cTnI double positive cells per view field. In A and D, sham mice were without ligation and treated with PKM2 (G415R) for 7 days. **(F) – (J)** Quantification of Masson Trichrome (F, G, and H) and WGA (I and J) staining of infarction region of myocardium from infarction mice that were treated with indicated agents. The tissue samples were collected at either 7 or 30 days post-infarction (MI or IR). The quantities are presented as % of Masson Trichrome ($M\text{-}T^{+}$, F, G, and H) or WGA (I and J) positive area per view field. **(K)** & **(L)** Representative images (K) or quantification (L) of α-SMA IHC staining of myocardium tissue of infarction region from IR infarction mice. The quantity is presented as % of α-SMA positive area per view field. The animals were euthanized 30 days after IR infraction. The animals were treated by the indicated agents. Error bars in (B), (C), (E), (F), (G), (H), (I), (J), and (L) represent mean ± S.E.M.. Scale bars in (A), 10 μm, (D) and (K), 100 μm. ns statistical no-significance, * $P > 0.05$, ** $P > 0.01$, *** $P > 0.005$, **** $P > 0.001$.

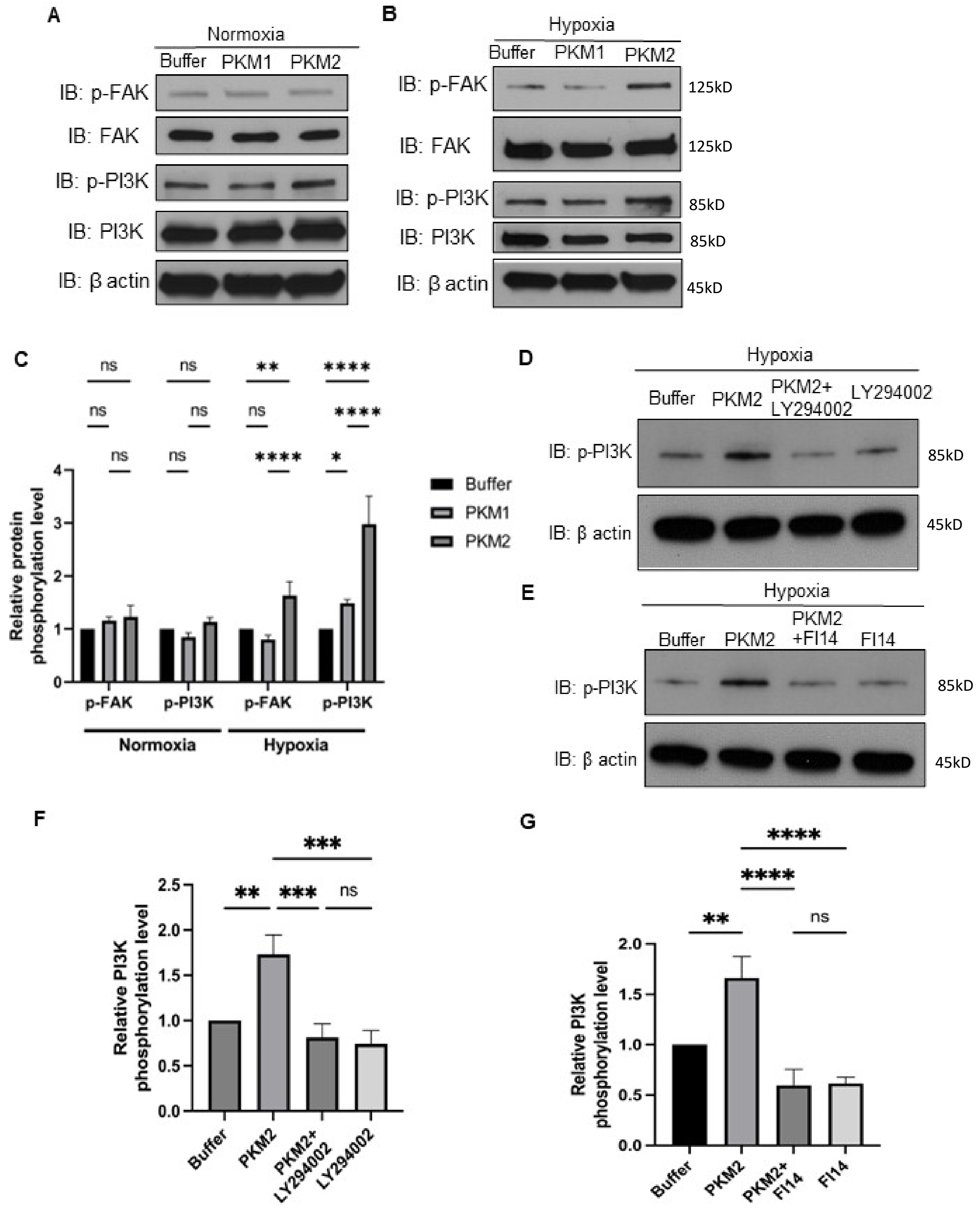

A
Normoxia
Buffer
PKM1
PKM2
IB: p-FAK
IB: FAK
IB: p-PI3K
IB: PI3K
IB: β actin
B
Hypoxia
Buffer
PKM1
PKM2
IB: p-FAK
IB: FAK
IB: p-PI3K
IB: PI3K
IB: β actin
125kD
125kD
85kD
85kD
45kD
C
Relative protein phosphorylation level
Buffer
PKM1
PKM2
p-FAK
p-PI3K
p-FAK
p-PI3K
Normoxia
Hypoxia
D
Hypoxia
Buffer
PKM2
PKM2+ LY294002
LY294002
IB: p-PI3K
IB: β actin
85kD
45kD
E
Hypoxia
Buffer
PKM2
PKM2 +FI14
FI14
IB: p-PI3K
IB: β actin
85kD
45kD
F
Relative PI3K phosphorylation level
Buffer
PKM2
PKM2+ LY294002
LY294002
G
Relative PI3K phosphorylation level
Buffer
PKM2
PKM2+ FI14
FI14

Figure. S7. EcPKM2 interacts with integrin $\alpha_v\beta_3$ and activates the integrin and downstream signaling.

(A) - (C) Cellular levels of phosphorylated p-FAK & p-PI3K in H9C2 cells that cultured under normoxia (A) and hypoxia (B) conditions and treated with indicated agents were analyzed by immunoblot and quantifications of four times independent immunoblots (C). In (A) and (B), immunoblots of FAK and PI3K are controls for total cellular levels of FAK and PI3K. In (C), the quantity of IB protein level is presented as relative level by define the measurements in buffer treated cells as 1. **(D) – (G)** Cellular levels of phosphorylated p-PI3K in primary human cardiomyocytes that cultured under hypoxia conditions and treated with indicated agents in the presence of PI3K inhibitor (D, LY294002) and FAK inhibitor (E, FI14) were analyzed by immunoblot and quantifications of four times independent immunoblots ((F) and (G)). In (F) and (G), the quantity of IB protein level is presented as relative level by define the measurements in buffer treated cells as 1. In (A), (B), (D), and (E), immunoblot of β-actin is a loading control. *The numbers on side of immunoblots in A, B, D, and E are molecular size markers*. Error bars in (C), (F), and (G) represent mean ± S.E.M.. ns statistical no-significance, * $P > 0.05$, ** $P > 0.01$, *** $P > 0.005$, **** $P > 0.001$.

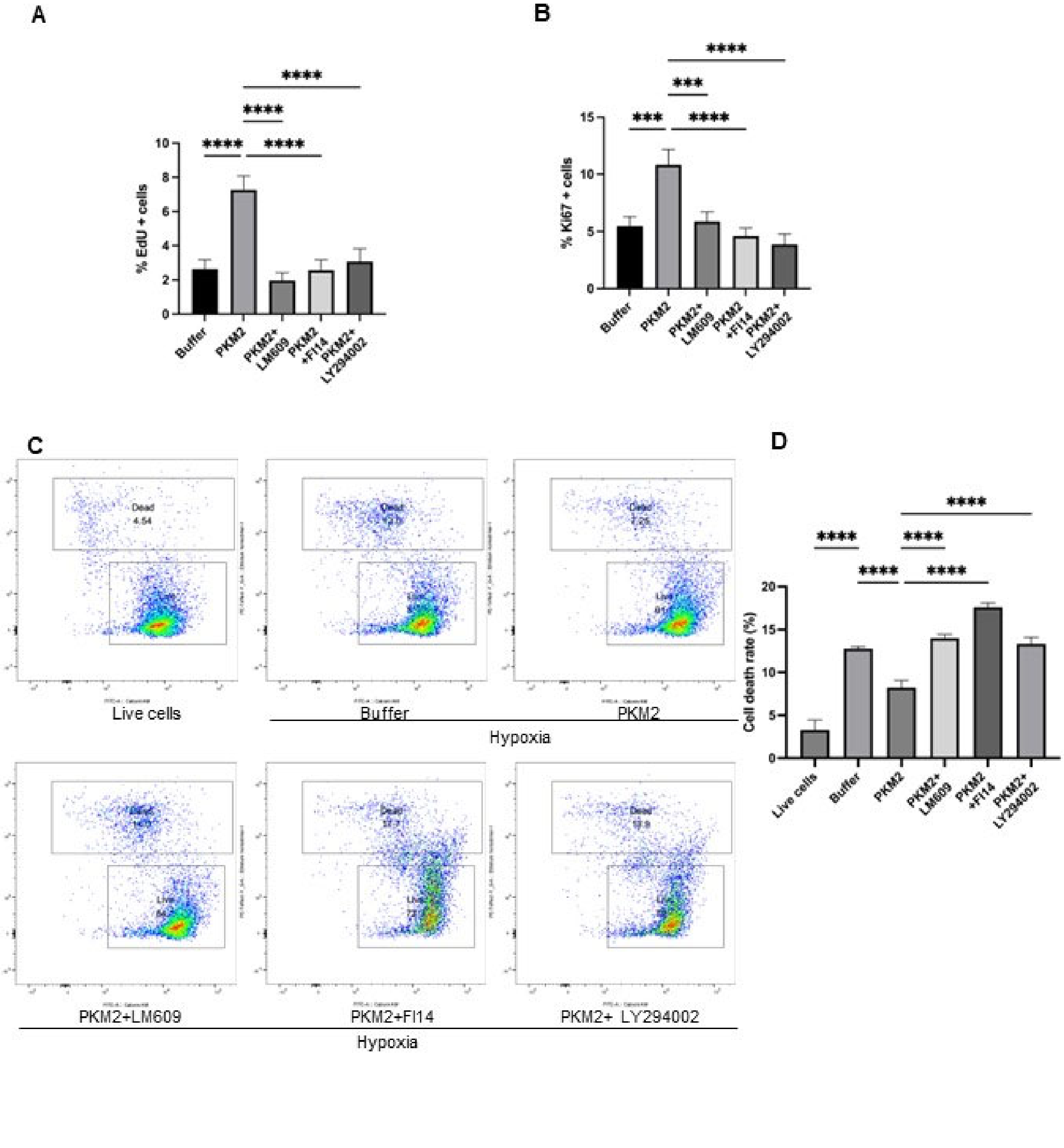

A
% EdU + cells
0
2
4
6
8
10
****
****
****
****
Buffer
PKM2
PKM2+ LM609
PKM2 +FI14
PKM2+ LY294002
B
% Ki67 + cells
0
5
10
15
***
***
****
****
Buffer
PKM2
PKM2+ LM609
PKM2 +FI14
PKM2+ LY294002
C
Dead
4.54
Live
Live cells
Buffer
PKM2
Hypoxia
PKM2+LM609
PKM2+FI14
PKM2+ LY294002
Hypoxia
D
Cell death rate (%)
0
5
10
15
20
****
****
****
****
****
Live cells
Buffer
PKM2
PKM2+ LM609
PKM2 +FI14
PKM2+ LY294002

Figure S8 EcPKM2 activates the integrin and downstream signaling protects cardiomyocytes from apoptosis and promotes proliferation.

(A) & **(B)** Quantification of EdU (A) and Ki67 IF staining (B) of H9C2 cells cultured under hypoxia conditions. The cultured cells were treated by the indicated agents (LM609, anti-integrin $\alpha_v\beta_3$ antibody, FI14, FAK inhibitor, LY294002, PI3k inhibitor). The quantities are presented as % of EdU (A) or Ki67 (B) positive cells in total cell population. **(C)** and **(D)** Cell death of H9C2 cells measured by FACS of Calcein AM and iodide staining. **(C)** Representative images of FACS profiles of cells under indicated treatments. **(D)** Quantification of FACS analysis. The quantification is presented as cell death rate (% of cells that are stained as dead cells in Calcein AM and iodide staining in total cell population). Live cells are experiment with the cells without treatment and under normoxia condition. Error bars in (A), (B), and (D) represent mean ± S.E.M.. *** P > 0.005, **** P > 0.001.
